# Bouquet Formation Failure in Meiosis of F_1_ Wheat–Rye Hybrids with Mitotic-Like Division

**DOI:** 10.3390/plants11121582

**Published:** 2022-06-15

**Authors:** Olga G. Silkova, Dina B. Loginova, Anastasia A. Zhuravleva, Vladimir K. Shumny

**Affiliations:** Department of Plant Genetics, Institute of Cytology and Genetics, Siberian Branch, Russian Academy of Sciences, Novosibirsk 630090, Russia; loginova.dina@gmail.com (D.B.L.); rogovaja_@mail.ru (A.A.Z.); shumny@bionet.nsc.ru (V.K.S.)

**Keywords:** wheat–rye amphihaploids, mitotic-like meiosis, telomeres, centromeres, subtelomeres, bouquet

## Abstract

Bouquet formation is believed to be involved in initiating homologous chromosome pairings in meiosis. A bouquet is also formed in the absence of chromosome pairing, such as in F_1_ wheat–rye hybrids. In some hybrids, meiosis is characterized by a single, mitotic-like division that leads to the formation of unreduced gametes. In this study, FISH with the telomere and centromere-specific probe, and immunoFISH with ASY1, CENH3 and rye subtelomere repeat pSc200 were employed to perform a comparative analysis of early meiotic prophase nuclei in four combinations of wheat–rye hybrids. One of these, with disomic rye chromosome 2R, is known to undergo normal meiosis, and here, 78.9% of the meiocytes formed a normal-appearing telomere bouquet and rye subtelomeres clustered in 83.2% of the meiocytes. In three combinations with disomic rye chromosomes 1R, 5R and 6R, known to undergo a single division of meiosis, telomeres clustered in 11.4%, 44.8% and 27.6% of the meiocytes, respectively. In hybrids with chromosome 1R, rye subtelomeres clustered in 12.19% of the meiocytes. In the remaining meiocytes, telomeres and subtelomeres were scattered along the nucleus circumference, forming large and small groups. We conclude that in wheat–rye hybrids with mitotic-like meiosis, chromosome behavior is altered already in the early prophase.

## 1. Introduction

Meiosis in eukaryotic organisms has two rounds of chromosome disjunction that follow a single round of DNA replication. The first disjunction involves homologous chromosomes; the second one involves sister chromatids. As a result, haploid gametes are formed, and their fusion restores the original ploidy in the next generation. 

An important feature of meiosis is that homologous chromosomes pair up for a proper reduction of the chromosome number, hence the efficient formation of valuable gametes. The core mechanisms that prepare the meiotic prophase nucleus to undergo the correct chromosome disjunction are widely conserved [1,2,3]. In a majority of organisms studied, homologous chromosome pairing is directly preceded in early leptotene by the clustering of telomeres to form the leptotene (or telomere) bouquet [3,4]. As synapsis first initiates near the telomeres [5,6,7], it has been proposed that the bouquet may help to facilitate pairing and synapsis.

During meiotic interphase, chromosomes have Rabl’s arrangement, a product of anaphase chromosome movement, where centromeres and telomeres occupy opposite poles of the nucleus. At the onset of the leptotene stage, prior to pairing, telomeres attach to the inner nuclear membrane [8]. During the leptotene/zygotene transition, the telomeres moving along the nuclear membrane form one cluster, the telomere bouquet [2]. Telomere movements are driven by the cytoskeleton that is connected to chromosomes through the components of the Linker of Nucleoskeleton and Cytoskeleton complex (LINC) [9,10,11].

The clustering density of telomeres in different species can vary from tight to loose, plus intermediate states [12]. To date, no plants without the bouquet structure were found in the studies [3,6,13,14]. The telomere dynamics at the early meiotic prophase is well known in such agricultural crops as maize (*Zea maize*) [8,15,16], barley (*Hordeum vulgare*) [17,18,19], rye (*Secale cereale*) [20,21,22,23] and wheat (*Triticum aestivum*) [7,19,24,25]. In barley, the telomeric bouquet clustering is completed in late G2; by leptotene, the telomeres are clustered [18], or the bouquet forms during leptotene, persisting during zygotene [17,19]. In rye, at early leptotene, a tight cluster is formed at the nuclear envelope [22,23]. In wheat, a fully formed bouquet with a tight telomere cluster is identified at early and mid-zygotene [7,19], despite the pairing in bread wheat being complicated by its hexaploid nature. Allohexaploid wheat (2*n* = 6× = 42) carries three homoeologous chromosome sets or subgenomes (A, B and D); however, crossing-over and chiasma formation occurs strictly between homologous chromosomes [26]. This diploid-like chromosome behaviour is controlled by the *Ph1* (*pairing homoeologous 1*) locus, which has been mapped on to the long arm of chromosome 5B [27,28,29]. 

Intergeneric wheat–rye F_1_ hybrids contain four haploid sets of chromosomes, three genomes of wheat (A, B and D), and one rye genome R, each with seven chromosomes, for the total of 28 chromosomes. With rare exceptions, there is no chiasma pairing in such hybrids as a result of the presence of *Ph1* [30,31,32,33], but telomeres still form the bouquet [33]. Studying the spread prophase nuclei using electron microscopy demonstrated that wheat–rye euploid hybrids form “bouquets” at mid-zygotene, but “bouquets” in hybrids with 5B nullisomy (*Ph1−*) are formed slightly earlier—during early zygotene [34]. In the other study, the telomere bouquet formation was shown at the onset of meiosis in both the presence and absence of *Ph1* [35]. Without chiasma pairing that would provide for regular chromosome segregation to gametes, wheat–rye hybrids are usually sterile. However, in some hybrids, functional gametes are formed that carry the somatic number of chromosomes [36,37,38]. The mechanism responsible for the formation of such gametes is known as meiotic restitution. In wheat–rye hybrids, meiosis is reduced to one division, which is similar to mitosis.

We have previously described distinct types of meiotic chromosome behaviour in wheat–rye hybrids (*2n* = 4× = 28, ABDR) with one pair of rye chromosomes present [38,39]. Among the four lines analysed, that with disomic rye chromosome 2R (in 2R(2D) substitution in the wheat parent) tends to promote reductional division in 83% of pollen mother cells (PMCs). In the absence of chiasma pairing, univalents randomly assort to the daughter nuclei in the first meiotic division, and separate sister chromatids in the second division. The resulting four products of these types of meiosis carry unbalanced chromosome numbers and constitutions, and in a great majority of cases are non-functional. Consequently, the hybrids are sterile. In contrast, in hybrids with disomic rye chromosomes 1R, 5R and 6R, the meiotic division is mitotic-like (more than 30%), where sister chromatids separate during the first division and there is no second one [38]. Hybrids are partly fertile and produce progeny with twice the chromosome number of the original hybrid. A distinguishing feature of the mitotic-like division is limited chromosome pairing (here, usually one homologous rye bivalent and occasional homologous wheat bivalents) or the complete absence of chromosome pairing [40,41,42]. These changes in the meiotic chromosome segregation may be associated with changes in the chromosome behavior in the early stages of the meiotic prophase. The telomere and rye subtelomere dynamics in the early meiotic prophase in wheat–rye hybrids has been extensively studied, but only in combinations with the standard (reductional) type of division. The primary focus of this research was to carry out a comparative analysis of telomere dynamics in the nuclei at the meiotic prophase in wheat, disomic substitution lines and in a set of the four wheat–rye hybrids described above and, specifically, to determine whether a telomere bouquet is formed in the hybrids with the mitotic-like meiotic division. 

## 2. Results

### 2.1. Telomere and Centromere Dynamics in Prophase in Bread Wheat Var. Saratovskaya 29, and Disomic Substitution Lines 1Rv(1A), 2R(2D) 

**Figure 1:**
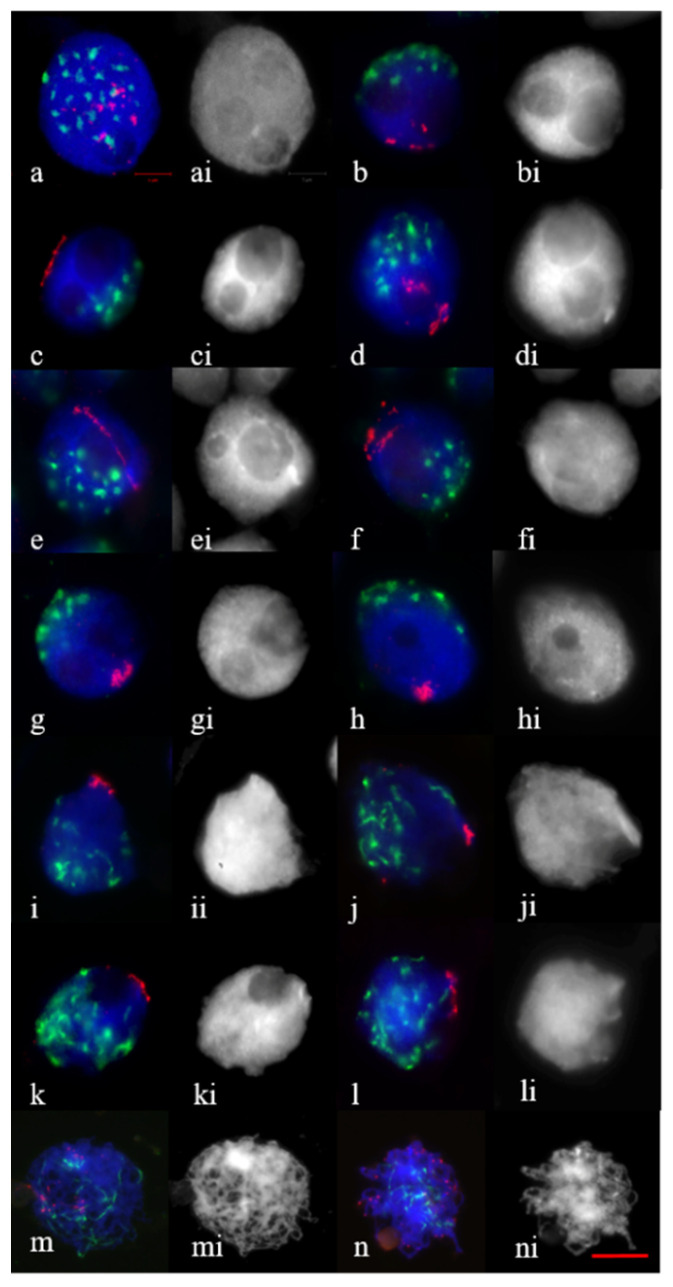
Telomere–centromere dynamics through meiotic prophase in wheat (**a**,**i**) and wheat–rye disomic substitution lines 1R(1A) (**c**,**e**,**g**,**k**,**m**), 2R(2D) (**b**,**d**,**f**,**h**,**j**,**l**,**n**). (**a**) Mid–late interphase with 3 nucleoli. (**b**–**h**) Leptotene with two nucleoli. Early–mid zygotene (**i**,**j**) with one peripherally located nucleolus. (**k**) Late zygotene, (**l**) early pachytene. (**m**) Pachytene. (**n**) Early diplotene. (**ai**–**ni**) The same as (**a**–**n**), DAPI. Telomeres are stained red and centromeres, green. Scale bar = 10 μm.

At interphase–zygotene transition, no difference was found in the analysis of telomere and centromere dynamics between Saratovskaya 29 and disomic substitution lines 1Rv(1A), 2R(2D). The meiocytes were studied starting from the onset of meiosis; at this time, 3–4 nucleoli could be observed in the nuclei (Figure 1a,ai). In the DAPI-stained PMC nuclei, nucleoli appeared as more or less spherical holes surrounded by a DAPI-positive chromatin (Figure 1ai–li). In meiocytes with three nucleoli, telomeres, single or in associations, were observed (Figure 1a). The centromeres aggregated in clusters of different sizes and shapes (Figure 1a). At leptotene, when the nucleoli fused to form two nucleoli, telomeres were seen to be in small or large clumps (Figure 1b,d,f) and occupied the arc of circumference with different lengths (Figure 1c,e). In meiocytes with two nucleoli, telomeres began to form clusters with different densities at the nuclear envelope (Figure 1g,h). These meiocytes were marked by centromeres associated in small groups at the periphery opposite to the telomere cluster (Figure 1g,h). At early zygotene, where one peripherally located nucleoli was found, telomeres associated into diffuse (Figure 1i) and tight clusters (Figure 1j); in centromere clustering, resolved elongated thinner centromeres occupied a nucleus hemisphere (Figure 1i,j). During the separation of the centromeres, the bouquet formation was fully completed. At late zygotene–pachytene, centromeres were distributed over the nucleus and telomere clusters’ disassembly occurred (Figure 1k–m). At early diplotene, telomeres were distributed in the form of points (Figure 1n).

### 2.2. Telomere and Centromere Dynamics during Prophase in Wheat–Rye F_1_ Hybrids with Different Meiotic Pathway

**Figure 2:**
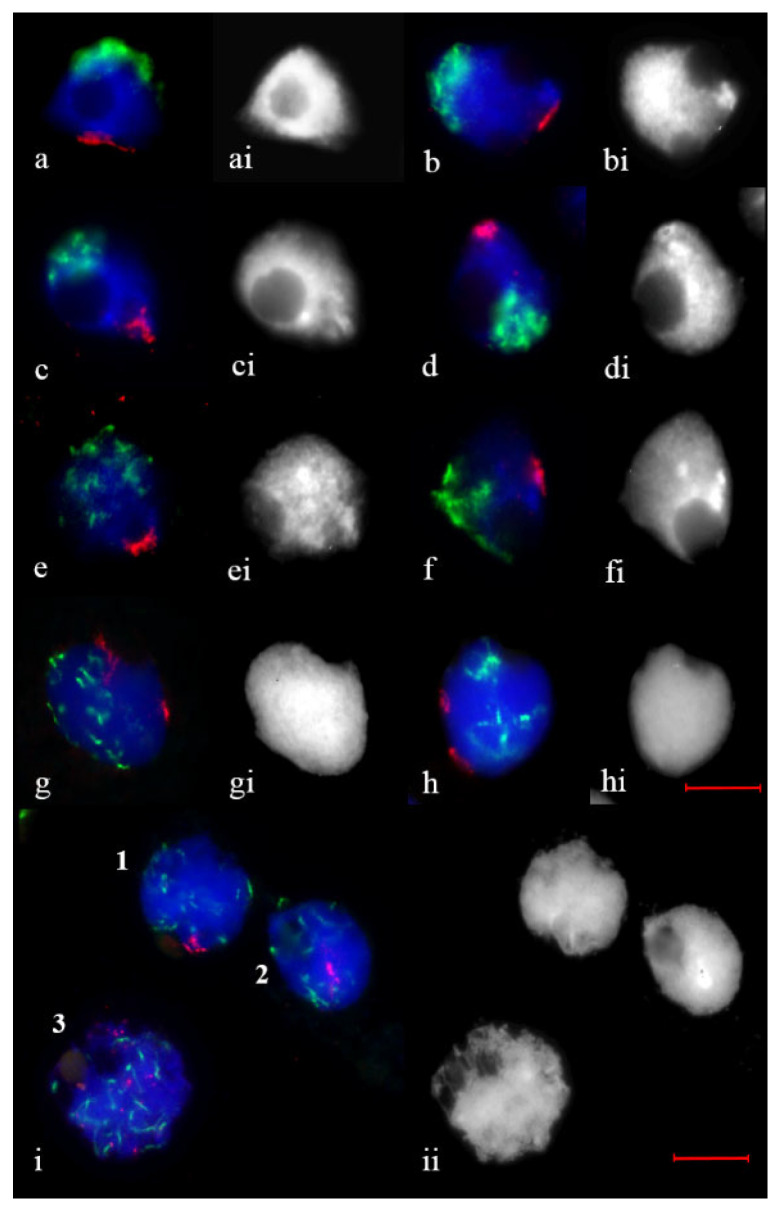
Telomere and centromere distribution in 2R(2D) × R hybrids. (**a**,**b**) Leptotene. (**c**,**d**) Early zygotene. (**e**–**h**) Zygotene. (**i**) Meiocytes at late zygotene (1, 2) and pachytene (3). Telomere bouquet is preserved (1) and resolved (2). Dispersed point telomeres and elongated centromeres are scattered within the nucleus (3). (**ai**–**ii**) the same as (**a**–**i**), DAPI counterstaining. Telomeres are stained *red*, centromeres, *green*. Scale bar = 10 μm.×.

In leptotene in 2R(2D) × R hybrids, centromeres gather into differently sized clusters and undergo polarization in a limited area of the nuclear periphery, and telomeres occupy part of a circular arc (Figure 2a,b). At early zygotene, telomeres are clustered. Half of all studied meiocytes had a single diffuse cluster (Figure 2c,e,f), and two diffuse clusters of unequal size (Figure 2g,h) formed in 21.1% of cells (Table 1). A single tight telomere cluster (Figure 2d) was found in 26.5% of cells (Table 1). Telomere clustering is relaxed at late zygotene; at pachytene, dispersed point telomeres and elongated centromeres are scattered within the nucleus (Figure 2i).

**Figure 3:**
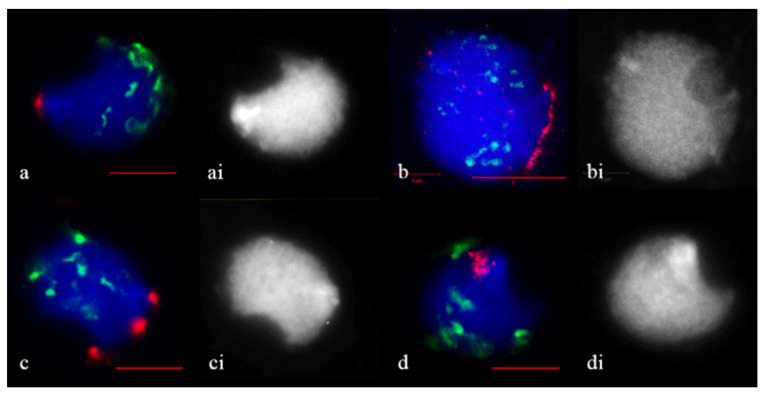
Telomere associations in 5R(5D) × R. Zygotene. (**a**) A tight telomere cluster. (**b**) Telomeres formed a part of circular arc. (**c**) Telomere clumps. (**d**) Diffuse telomere cluster. (**ai**–**di**) the same as (**a**–**d**), DAPI counterstaining. Telomeres are stained *red*, centromeres, *green*. Scale bar = 10 μm.

In the 5R(5D) × R hybrids, 44.8% of the meiocytes had tight (Figure 3a) and diffuse (Figure 3d) telomere clusters at early zygotene (Table 1). In the remaining meiocytes, the telomeres formed small and large clumps (32.2%) (Figure 3c) or occupied the arc of the nucleus circumference (23%) (Figure 3b). The centromeric regions were located in the nucleus hemisphere in meiocytes where tight telomere cluster formed (Figure 3a) or centromeres were distributed around the nucleus in the other cases (Figure 3b–d).

**Figure 4:**
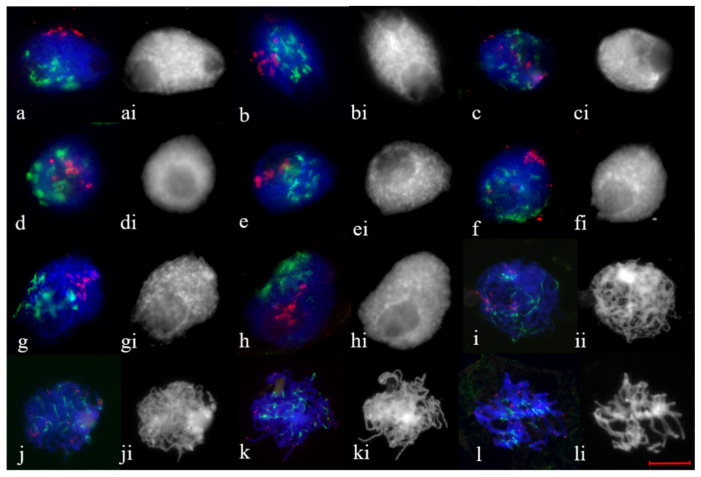
Telomere and centromere dynamics in 1R(1A) × R (**a**,**c**,**e**–**h**,**j**,**l**) and 6R(6A) × R (**b**,**d**,**i**,**k**) hybrids. (**a**,**b**) Leptotene. (**c**–**h**) Zygotene. (**i**,**j**) Pachytene, (**k**,**l**) Diplotene. (**ai**–**li**)—the same as (**a**–**l**) DAPI counterstaining. Telomeres are stained *red*, centromeres, *green*. Scale bar = 10 μm.

In the 1Rv(1A) × R and 6R(6A) × R hybrids, telomere associations in the early stages of prophase were similar, but were substantially different relative to wheat and the 2R(2D) × R hybrids. At leptotene, in meiocytes with two nucleoli, centromeres formed diffuse structures, and telomeres associated into large or small groups (Figure 4a–c); in some meiocytes these groups localized along the nucleus circumference (Figure 4a,c). During the leptotene-to-zygotene transition and at early zygotene, telomere associations represented groups of various shapes and sizes (Figure 4d–i), clumps localized along the nucleus circumference (Figure 4d,h) or the telomeres-formed cluster (Figure 4j). There was no clear polar arrangement of telomeres and centromeres, and the centromeres looked like a diffuse elongated structure in the overwhelming majority of meiocytes in which telomere clusters were not found (Figure 4e–i). In the meiocytes, where clusters of telomeres were found, the polar arrangement of centromeres and telomeres was preserved (Figure 4j). In general, an analysis of telomere associations in the 1Rv(1A) × R hybrids shows that clustering of the telomeres occurred in 11.4% of the nuclei at early zygotene (Table 1). Telomeres localized along the nucleus circumference in 28.2% of the meiocytes or formed several large or small clumps in 60.4% of the meiocytes (Table 2). Similar associations of the telomeres were found in the 6R(6A) × R hybrids. However, more meiocytes with telomere clusters (27.6%) and fewer meiocytes with telomeres located along the nucleus circumference (8.3%) were found in the 6R(6A) × R hybrids than in the 1Rv(1A) × R hybrids (Table 1). 

### 2.3. Subtelomere of Rye and Centromere Dynamics during Prophase in Wheat–Rye F_1_ Hybrids with Different Meiotic Pathway

**Figure 5:**
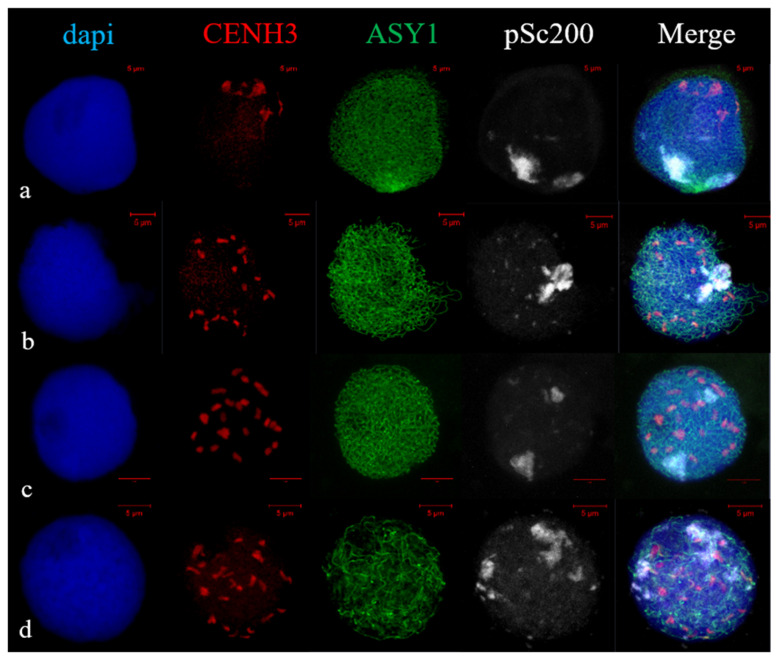
CENH3 and pSc200 behaviour at leptotene-to-pachytene stages, marked ASY1 loading, in 2R(2D) × R hybrids. (**a**) Leptotene. CENH3 indicates centromere clustering, continuous ASY1 signal at restrict area, subtelomeres began to associate in a small area. (**b**–**d**) Zygotene. ASY1 signals are visible along chromatin axes. Centromeres distributed over nucleus. Subtelomeres are associated in single cluster (**b**), in two clusters (**c**). (**d**) Pachytene. Initiation of ASY1 disassembly, subtelomere distribution. ImmunoFISH. Confocal image, maximum intensity projection. Scale bar = 5 μm.

The arrangement of the rye chromosome subtelomeres at early stages of meiotic prophase using immunoFISH with ASY1, CENH3 and pSc200 rye subtelomere repeat probe was studied. We analyzed meiocytes of 1R(1A) × R and 2R(2D) × R hybrids at leptotene-to-pachytene. At leptotene in 2R(2D) × R hybrids, ASY1 was localized on the chromatin axis in the form of short segments, followed by their elongation (Figure 5a). The area of short ASY1 segments coincided with the localization of pSc200 repeats. Centromeres, single or combined, were located polarly relative to pSc200 in a limited area of the nuclear periphery. During zygotene the ASY1 signal elongated, and CENH3 signals were dispersed throughout the nucleus (Figure 5b,c). Different types of rye subtelomere associations were observed. Subtelomeres formed one cluster in 83.2% of meiocytes (Figure 5b) or two clusters in 16.8% of meiocytes (Figure 5c, Table 2). During pachytene, the dissociation of ASY1 and distribution of rye subtelomeres along the nucleus began (Figure 5d).

**Figure 6:**
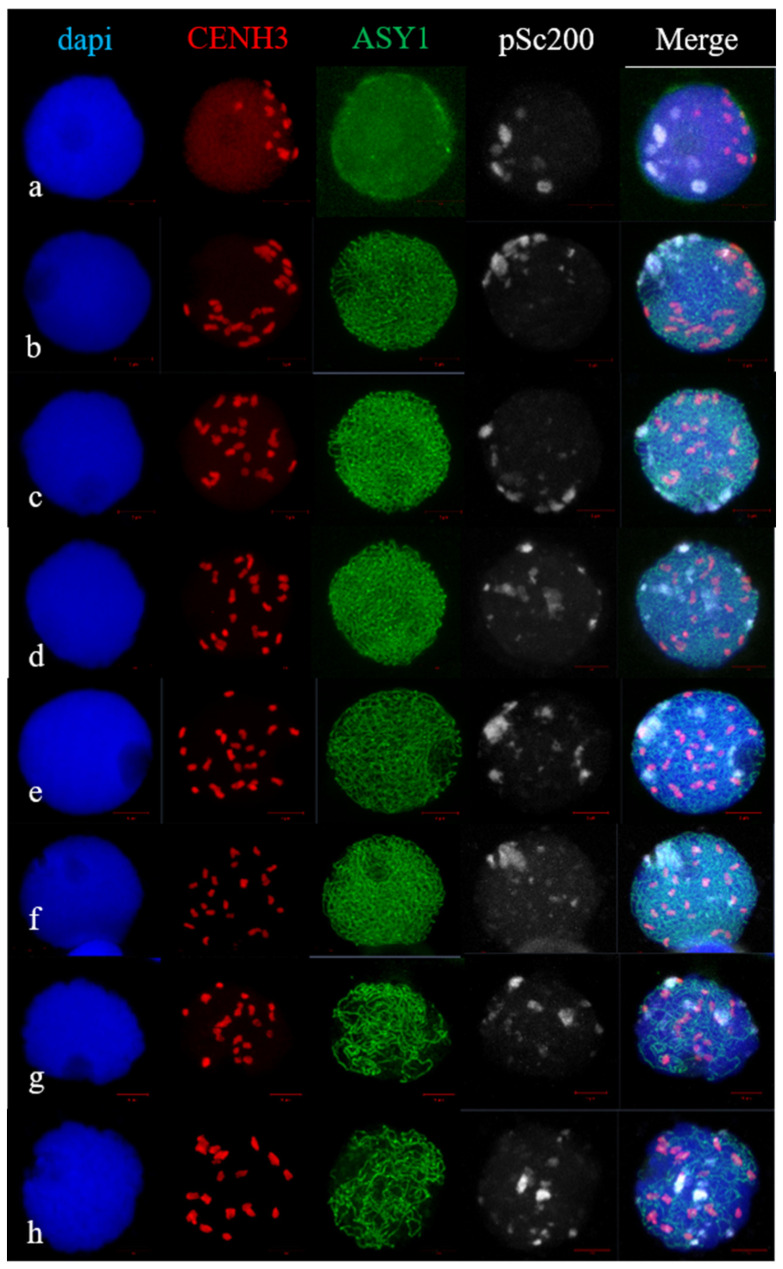
CENH3 and pSc200 behaviour at leptotene-to-pachytene stages, marked by ASY1 loading in 1R(1A) × R hybrids. (**a**) Leptotene. CENH3 indicates centromere clustering, ASY1 signals form part of circular arc, subtelomeres form clumps and occupy a circular arc. (**b**–**f**) Zygotene. ASY1 signals are visible along chromatin axes. (**b**) Centromeres and subtelomeres are polarized and occupy circular arcs. (**c**) Centromeres distributed over nucleus, subtelomeres formed part of circular arc. (**d**,**e**) Large and small subtelomere clumps as well as centromeres distributed over nucleus. (**f**) Centromeres distributed over nucleus, subtelomeres are associated in cluster. (**g**) Late zygotene–early pachytene. Initiation of ASY1 disassembly, subtelomeres distributed over the nucleus. (**h**) Pachytene. ASY1 disassembly, subtelomeres distributed over the nucleus. ImmunoFISH. Confocal image, maximum intensity projection. Scale bar = 5 μm.

At leptotene, 1R(1A) × R hybrids had an association of centromeric regions; they were located polarly with respect to rye subtelomeres. ASY1 signals appeared, but no characteristic localization in the form of segments was found in a limited area of the nuclear periphery (Figure 6a). The brighter point ASY1signals were distributed around the circumference of the nucleus. At the zygotene stage, the ASY1 signal elongated, and the CENH3 signals were dispersed throughout the nucleus (Figure 6b–f). Using pSc200 labeling, we observed different types of rye subtelomeric associations. Subtelomeres formed one cluster in 12.19% of meiocytes, two clusters in 9.75% of meiocytes, clumps in 65.87% of meiocytes and were localized along the nuclear circumference in 12.19% of meiocytes (Figure 6b–f, Table 2). At late zygotene and pachytene, the dissociation of ASY1 began, and the distribution of rye subtelomeres along the nucleus was preserved (Figure 6h). Thus, the types of rye subtelomeric associations in 1R(1A) × R and 2R(2D) × R hybrids corresponded to the types of telomeric associations obtained using FISH analysis.

## 3. Discussion

### 3.1. Defining the Time-Course of Early Prophase Progress in Wheat and Wheat–Rye Hybrids

To obtain proper results, defining leptotene and zygotene in bread wheat and wheat–rye hybrids was an essential element of our study. We used such criteria as the number and position of nucleoli in meiocyte nuclei [43,44], character of ASY1 localization, distribution and share of centromeres [17,18,19], and the cell shape [21] described for wheat, barley and rye. In the course of meiocyte development, the nucleoli fuse (reunite) into one before or during leptotene [43,44]; as a result, during the leptotene-to-zygotene transition, one large nucleolus localizes on the nucleus periphery [7,8,21,22,44,45]. For both wheat and rye, full telomere aggregation coincides with nucleoli fusion [19,21,24,25,44]. According to Sepsi et al. [19], in the mid–late interphase of bread wheat, ASY1 appeared in meiocyte nuclei with 2–4 nucleoli; by leptotene, ASY1 formed continuous thin threads along the whole length of the chromosomes and shot bright stretches in the restricted area near the nuclear envelope. An important feature is the location and structure of the centromeres. At leptotene, centromeres, marked with CENH3, underwent a polarization, and, at that time, centromeres were seen as highly compact, point-like structures; at zygotene, centromeres were seen as significantly expanded elongated threads and localized in the nuclear hemisphere [19]. In the course of the centromere dispersion, the telomere bouquet was fully formed with tight telomere clustering [19]. At the zygotene stage of rye, intense ASY1 signals were visible along the chromatin axes. When a synapse occurs at pachytene, the ASY1 signal becomes diffuse and has a significantly lower fluorescence intensity [46].

### 3.2. Bouquet Formation in Wheat and Wheat–Rye F1 Hybrids with Different Meiotic Pathway

The bouquet is a specific conserved configuration of meiosis concomitant with important events of homologue juxtaposition that ensured the correct chromosome segregation. Several features are common to all known bouquets: telomere regions are associated with the nuclear envelope, grouped within a restricted area, and move in and out of the bouquet at particular stages of meiosis, the leptotene/zygotene transition, and into early pachytene [47]. The clustering density of telomeres in different species can vary from tight to loose, plus intermediate states [12]. To date, no plants without the bouquet structure have been found in studies [4,13,14,16]. The clustering of telomeres is coincident with the initiation of synaptonemal complex (SC) development [4,7,19], although pairing and bouquet formation are mutually independent [4,47]. It is not surprising that the bouquet is observed in wheat–rye hybrids [34,48,49], in which genomes do not have homologues and in which bivalents are formed in individual cases. According to electronic microscopy data, telomere clustering in hybrids occurs at early zygotene and is maintained until late zygotene, which is the period when long SC tracks are formed from telomeres [34].

In this study, at the onset of meiosis when there were 3–4 nucleoli in the nuclei, the localization of axial protein ASY1 was initiated. It appeared that meiotic structure is typical for chromatin in hybrids, similarly to wheat and disomic substitution lines, since the specific protein of meiotic axial element ASY1 was observed. At that time, single telomeres and small telomeric clumps were observed, and the centromeres formed different sized and shaped associations in wheat, substitution lines and wheat–rye hybrids. It should be noted that the location of the centromere and telomere was polar. As prophase progressed, at leptotene, in wheat meiocytes with two nucleoli, telomeres formed diffuse clusters and centromere associations remained polarized. At early zygotene, diffuse and tight clusters were found. It is likely that the diffuse cluster preceded the tight cluster; however, it is rather difficult to address the issue clearly because tight and diffuse clusters formed in meiocytes with two nucleoli and those meiocytes were visually similar. Bouquets with different telomere densities are not described in wheat [7,19], but different patterns of telomeres’ clustering have been found in Arabidopsis, which has great variability in the number of telomeres in a bouquet and its volume [14]. In addition, it has been shown that clustering density depends on the prophase stage; in filamentous fungus *Sordaria macrospora*, a loose bouquet was found at leptotene–zygotene and a tight bouquet at pachytene [12]. Further research will help determine the cause of the different clustering densities in wheat and lines found in this study.

The hybrids analysed in this study demonstrated different patterns of telomere clustering. For the 2R(2D) × R hybrids, 78.9% of the meiocytes formed the classic bouquet structure with a diffuse or tight telomere cluster and centromeres distributed in the nucleus hemisphere. A special feature of those hybrids was that they formed two telomere clusters of unequal size in 21.1% of the meiocytes. Conversely, the majority of meiocytes in the 5R(5D) × R, 1Rv(1A) × R and 6R(6A) × R hybrids did not have the classic bouquet structure. The telomeres in these meiocytes were associated in large and small clumps or located along the nucleus circumference; the polarization of centromeres vs. telomeres in the nucleus was absent. 

At the onset of meiotic prophase in rye, the subtelomeric regions in PMCs cluster into a typical bouquet conformation [22]. ImmunoFISH with ASY1, CENH3 and rye pSc200 repeat probe allowed us to make sure that the 2R(2D) × R hybrids formed a cluster of subtelomeres, corresponding to the bouquet structure. Meiocytes with one peripherally located nucleolus, elongated ASY1 strands and dispersed CENH3 signals were clearly at the zygotene stage. However, in meiocytes of 1Rv(1A) × R hybrids, different types of rye subtelomere associations were found. They were identical to those in the analysis using FISH in both hybrids. 

A similar telomere distribution was discovered in synaptic mutants of maize—*pam1* [15] and rye—*sy1* [22]; a telomere misplacement phenotype during the bouquet stage was observed in maize desynaptic *dy* and *dsy1* mutants [50,51]. The cause for the disruption in the formation of the bouquet in *dy* and *dsy1* mutants is a defect in telomere–nuclear envelope interactions. For these interactions and telomere clustering, the meiotic ZmSUN2 protein is needed [52]. Immunolabelling showed that ZmSUN2 located to a nuclear periphery as a belt-like structure at the leptotene, changed to a half-belt during zygotene, and telomeres were localized inside the SUN2 half-belt [52]. In meiosis-specific mutants *dy1*, *as1* and *dv1*, the disruption of the meiotic SUN2 belt was observed; this fact provides additional evidence for the role of the nuclear envelope in meiotic chromosome behavior [52]. Perhaps, the meiotic SUN belt serves as an attachment platform for limitation of the telomere motility within a restricted zone on the nuclear envelope [52]. In rice (Oryza sativa), cytological analyses of the double mutant *OsSUN1OsSUN2* revealed severe defects in telomere clustering, homologous pairing and crossover (CO) formation [53].

In certain organisms telomeres move in and out of the bouquet at particular stages of prophase, whereas in others, such as in *S. pombe*, telomeres remain tightly clustered during prophase [12,47]. Rapid, short-distance oscillations of small individual chromosome segments extending from the main chromatin mass into the nuclear space, which were mostly telomere-led, have been reported in live maize meiotic cells at zygotene [54]. Obviously, telomere movements at zygotene should be completed or restricted by the time ZYP1 is localized in the telomere clustering region [17,18,19]. Nevertheless, we found only 11.4% of meiocytes with a telomere cluster in 1Rv(1A) × R hybrids at zygotene.

The movement of telomeres is facilitated by their interaction with cytoskeleton structures involving SUN and KUSH proteins [12]. However, plants do not show the direct participation of the cytoskeleton in the movement of telomeres [55]. In maize, telomeres were localized inside the SUN2 half-belt, but neither F-actin nor microtubules were observed to form structures related to this belt [52].

In summary, we can assume that the lack of telomere clustering within a restricted area on the nuclear envelope during zygotene in most meiocytes in 5R(5D) × R, 1Rv(1A) × R and 6R(6A) × R hybrids may be caused by the failure of telomeres to make contact with the inner nuclear envelope, perhaps due to failure in the functioning of SUN proteins.

## 4. Materials and Methods

### 4.1. Plant Material

This study used wheat *T. aestivum* L. cv. Saratovskaya 29 (designated as S29, BBAADD, 2*n* = 42), and its four single rye chromosome substitutions 1R(1A), 2R(2D), 5R(5D) and 6R(6A), as described before [56,57]. Rye chromosomes 1R originated from *S. cereale* cv. Vyatka; chromosomes 2R, 5R and 6R from cv. Onokhoinskaya. These wheat lines were pollinated with diploid rye cv. Onokhoiskaya (RR, *2n* = 14), to create 28-chromosome F_1_ hybrids, hereafter called 2R(2D) × R, 1Rv(1A) × R, 5R(5D)x, and 6R(6A) × R. The F_1_ hybrids, wheat and rye plants were grown under greenhouse conditions at a temperature of 24/18 °C day/night and under a day/night cycle of 16/8 h.

### 4.2. FISH and Immunostaining on Meiotic Chromosomes

For FISH, spikes were fixed in 45% acetic acid for 2–4 h at room temperature, anthers with meiocytes at interphase–pachytene were selected and squashed with one anther per slide. Slides were frozen in liquid nitrogen, coverslips removed and preparations dehydrated through ethanol series, at concentrations of 70%, 90% and 96%, and stored at −20 °C until needed. Each anther was examined individually, and all scorable PMCs were assayed (Table 3). Telomeric regions of all chromosomes were visualized with digoxigenin-11-dUTP-labelled telomere-specific probe (tandem repeat CCCTAAA). Probe labelling was by PCR according to Cheung et al. [58]. Centromeres were labelled with probe pAet6-09 originating from *Aegilops taushii*. This probe indiscriminately labels centromeres of rye, wheat, rice and barley chromosomes [59,60]. The probe was provided by Dr. A. Lukaszewski (University of California, Riverside, CA, USA). Probe labelling was performed using the Nick Translation System (Invitrogen, Carlsbad, CA, USA, cat. no. 18160-010) with biotin 16-dUTP. Two probes were used in combination and were mixed with blocking wheat DNA. Chromatin was stained using 1 mg/mL DAPI in Vectashield anti-fade solution (Vector Laboratories, Burlingame, CA, USA). In situ hybridization with labelled DNA probes was performed according to Silkova and Loginova [38].

The combination of immunostaining with FISH was used according to Schubert et al. [61]. Primary rabbit antibodies against ASY1 (1:150; Agrisera, Vännäs, Sweden; cat. no. AS08 323) and direct-labelled (NHS-Rhodamine, Cat Number 46406) rabbit anti-CENH3 (1:800; kindly provided by Dr. A. Houben, IPK, Gatersleben) were used to visualize time-course of prophase stages. ASY1 is a marker for chromosome axis, CENH3 is centromere-specific histone. For detection, goat anti-rabbit rhodamine (1:100; Thermo Fisher Scientific, Waltham, MA, USA; cat. no. 31670) and goat anti-rabbit Alexa Fluor 488 (1:100; Jackson Immunoresearch, West Grove, PA, USA; cat. no. 111-545-003) were applied. Subtelomeric rye repeats pSc-200 [62] for FISH were directly labelled by nick translation using an Atto647N NT Labelling Kit (Jena Bioscience, Jena, Germany; cat. no. PP-305S-647N).

The method reported by Manzanero et al. [63] was used with slight variations. Anthers were fixed in fresh 8% paraformaldehyde in phosphate-buffered saline (PBS) for 2 h in a humid chamber, washed 4 × 15 min in PBS, and digested at room temperature for 5 to 15 min in a mixture of 1% pectinase, 1% cellulase Onozuka R-10 and 1% pectolyase Y-23 dissolved in PBS.

The material was disaggregated on poly-L-lysine-coated slides. After freezing for 15 min at −70 °C and blocking for 30 min in 3% bovine serum albumin (BSA)/PBS/non-fat milk, incubation with the primary antibodies was completed overnight at 4 °C. Then, slides were washed 4 × 15 min in PBS and incubated with the secondary antibody at room temperature for 1 h. After 4 × 15 min washes in PBS, the slides were counterstained with 4′,6-diamidino-2-phenylindole (DAPI) and mounted in anti-fade Vectashield medium.

Prior to FISH the slides were treated with ethanol–acetic acid (3:1) fixative for 10 min and freshly prepared 4% formaldehyde in 1 × PBS for 10 min, followed by three times washing for 5 min in 1 × PBS [63]. These steps are important to stabilize the immunosignals during the following FISH procedure, which was performed as described above.

Slides were examined under an Axio Imager M1 (Carl Zeiss AG, Oberkochen, Germany) microscope, images were recorded with a ProgRes MF camera (Meta Systems, Jenoptic, Jena, Germany) and Isis software (Meta Systems) and processed using the Adobe Photoshop software version 12.0.4 (Adobe Design Std CS5 5.0 WIN AOO License RU (65057900). Immunolabelled slides were examined under a confocal laser scanning microscope LSM 780 NLO (Zeiss) with a monochrome digital camera AxioCam MRm (Zeiss) and ZEN software (Zeiss).

## 5. Conclusions

Wheat–rye hybrids are a valuable material to study the regulation of developing unreduced gametes. The use of wheat–rye hybrids enables the study of the regulation of meiosis in plants with a polyhaploid genome. Here, new data showed that most meiocytes in hybrids with mitotic-like meiosis lack the meiosis-specific bouquet structure; telomeres form various association patterns: located along the nucleus circumference and the point foci, and in large and small clumps.

## Figures and Tables

**Table 1:** Percent of meiocytes with different types of telomere associations at zygotene in wheat–rye F_1_ hybrids (FISH).

Hybrids	Tight Cluster	One Diffuse Cluster	Two Diffuse Clusters	Clumps	Location along the Nucleus Circumference
2R(2D) × R	26.5	52.4	21.1	0	0
1R(1A) × R	6.3	5.1	0	60.4	28.2
5R(5D) × R	8.6	36.2	0	32.2	23
6R(6A) × R	17.4	10.2	0	64.1	8.3

**Table 2:** Percent of meiocytes with different types of rye subtelomere associations at zygotene in wheat–rye F_1_ hybrids (ImmunoFISH).

Hybrids	Cluster	Two Clusters	Clumps	Location along the Nucleus Circumference
2R(2D) × R	83.2	16.8	0	0
1R(1A) × R	12.19	9.75	65.87	12.19

**Table 3:** Materials analysed in the study.

	FISH			Immunostaining + FISH	
	Plants	Anthers/Slides	Meiocytes	Plants	Meiocytes
2R(2D) × R	5	14	863	3	97
1R(1A) × R	5	16	1637	5	41
5R(5D) × R	3	8	476	-	-
6R(6A) × R	3	9	661	-	-
S29	3	9	353	-	-
2R(2D)	5	10	756	-	-
1R(1A)	5	16	1036	-	-

## Data Availability

Data are contained within the article.
